# Tangeretin has anti-asthmatic effects via regulating PI3K and Notch signaling and modulating Th1/Th2/Th17 cytokine balance in neonatal asthmatic mice

**DOI:** 10.1590/1414-431X20175991

**Published:** 2017-07-17

**Authors:** L.-L. Liu, F.-H. Li, Y. Zhang, X.-F. Zhang, J. Yang

**Affiliations:** 1 Children's Medical Center, Qilu Hospital of Shandong University, Jinan, Shandong, China; 2 Department of Pathology, Shandong University of Medicine, Jinan, Shandong, China

**Keywords:** Asthma, Inflammation, Notch signaling, PI3K, Tangeretin, T-helper cells

## Abstract

Asthma is a chronic allergic disease characterized by airway inflammation, airway hyper-responsiveness (AHR), and mucus hypersecretion. T-lymphocytes are involved in the pathogenesis of asthma, mediating airway inflammatory reactions by secreting cytokines. The phosphoinositide 3-kinase (PI3K) and Notch signaling pathways are associated with T cell signaling, proliferation, and differentiation, and are important in the progression of asthma. Thus, compounds that can modulate T cell proliferation and function may be of clinical value. Here, we assessed the effects of tangeretin, a plant-derived flavonoid, in experimental asthma. BALB/c mice at postnatal day (P) 12 were challenged with ovalbumin (OVA). Separate groups of mice (n=18/group) were administered tangeretin at 25 or 50 mg/kg body weight by oral gavage. Dexamethasone was used as a positive control. Tangeretin treatment reduced inflammatory cell infiltration in bronchoalveolar lavage fluid (BALF) and also restored the normal histology of lung tissues. OVA-specific IgE levels in serum and BALF were reduced. AHR, as determined by airway resistance and lung compliance, was normalized. Flow cytometry analyses revealed a reduced Th17 cell population. Tangeretin reduced the levels of Th2 and Th17 cytokines and raised IFN-γ levels. PI3K signaling was inhibited. The expressions of the Notch 1 receptor and its ligands Jagged 1 and 2 were downregulated by tangeretin. Our findings support the possible use of tangeretin for treating allergic asthma.

## Introduction

Asthma, an airway inflammatory disease, is common, chronic, and increasing in prevalence. It is associated with an extensive array of symptoms that include mucus hypersecretion, airway hyper-responsiveness (AHR), and airway remodeling (1). T lymphocytes play major roles in airway inflammation and remodeling through cytokines (2). Th2 cytokines (IL-4, IL-5, IL-13) induce allergen-specific immunoglobulin (Ig) E production and inflammatory mediator release from mast cells (2). IFN-γ, secreted by Th1 cells, suppresses Th2 immune responses. Class switching is induced by IL-4 in IgG1 and IgE, whereas IFN-γ is associated with IgG2 α class switching (3). Thus, Th1/Th2 cytokine stability is an important measure in the assessment of asthma (3).

Th17 cells, a subset of T lymphocytes, have been reported to be involved in the pathogenesis of several immune-mediated disorders and also in asthma (4). Th17 cells release several cytokines, including IL-17, IL-6, TNF-α, and IL-22. IL-17 induces eosinophil infiltration and the recruitment of macrophages (5) in asthma models. Studies have shown an increase in Th17 cells in airway inflammation in asthma (6). Thus, regulation of Th17 cells could be of benefit in asthma therapy.

Phosphoinositide 3-kinase (PI3K) is a major enzyme involved in cell proliferation, differentiation, and cell signaling. On activation, PI3K activates its effector protein Akt, that in turn affects several down-stream targets as mammalian target of rapamycin (mTOR) and glycogen synthase kinase 3β (GSK3β). PI3K/Akt pathway is negatively regulated by phosphatase and tensin homolog (PTEN) (7). PI3K is widely distributed in lung tissue and plays an important role in T cell signaling (8). In particular, PI3K activation in T cells promotes survival (9) and cell cycle progression (10), modulates differentiation and controls the acquisition of effector and memory phenotypes (11). The pathway critically influences the inflammatory response of the airway (12). Thus, inhibitors of PI3K/AKT/mTOR pathway can interfere with T cell activation and function, and would be beneficial in inflammatory disorders. Herrero-Sánchez et al. (13) reported that the targeted inhibition of the PI3K/Akt pathway effectively suppressed T cell activation and prevented graft-versus-host disease development.

The Notch signaling pathway is also involved in T cell activation and differentiation (14). Dysregulation of Notch signaling may induce human disorders such as asthma. Pharmacological inhibitors of Notch signaling can regulate Th1 and Th2 cell-mediated inflammatory responses, reducing allergic pulmonary inflammation (15). Notch signaling regulates retinoic acid-related orphan receptor *γt*, an important transcription factor in Th17 differentiation (16). Thus, inhibition of Notch signaling may be beneficial to treat asthma, and compounds that regulate PI3K signaling may be of therapeutic value.

In this study, we assessed the anti-asthmatic effects of tangeretin, an O-polymethoxylated flavone found in tangerine and other citrus fruits. Tangeretin exhibits significant anti-cancer, anti-oxidant and anti-proliferative, activities (17). Studies have reported that tangeretin inhibits inflammatory responses by inhibiting NF-κB activation and pro-inflammatory cytokines as interleukins (IL-1β) and tumor necrosis factor (TNF-α) (6,17,18). In this study, we assessed the ability of tangeretin to modulate PI3K/Akt and Notch signaling and Th2/Th1 and Th17 cytokine levels in experimentally induced asthma in neonatal mice, considering the various biological activities of tangeretin.

## Material and Methods

### Chemicals and antibodies

Ovalbumin (OVA), methacholine, dexamethasone (DEX), tangeretin, and modified Wright-Giemsa stain were from Sigma-Aldrich (USA). ELISA (Enzyme-linked immunosorbent assay) kits for IL-4, IL-5, and IL-13 were obtained from Biolegend (USA). Antibodies against cyclinA, cyclinD1, Akt, p-Akt, GSK-3β, p- GSK-3β, phosphatise, and PTEN were from Cell Signaling Technology (USA). Mammalian target of rapamycin (mTORc1), Jagged 1, Jagged 2, Notch 1, Notch receptor intracellular domain (NICD), and p27kip1 antibodies were from Santa Cruz Biotechnology (USA). FITC-labelled anti-human CD4, phycoerythrin (PE), and anti-mouse IL-17A were from eBioscience Co. (USA). All other analytical-grade chemicals were from Sigma-Aldrich unless noted otherwise.

### Experimental animals

BALB/c mice were obtained from Shandong University Experimental Animal Centre (China) and were maintained in sterile cages in a controlled environment (24±1°C, 40–80% humidity) with a 12/12 h day/night cycle and were provided with water and food *ad libitum*. The animals were carefully monitored for the day of birth of pups, which was noted as postnatal day 0 (P0). This study was approved by the Ethical Committee of the Shandong University (SD2015031428). Study animals were maintained and handled according to international regulatory guidelines for the maintenance of experimental laboratory animals (19).

### Experimental design

P12 mice (n=18/group) were used. Separate groups of mice were challenged with OVA after sensitization, as described by Bao et al. (20). Mice on days 0 and 14 were sensitized with *ip* injections of OVA (20 μg) and 4 mg Al(OH)_3_ suspended in saline (0.1 mL). Furthermore, they were tested with 1% OVA aerosol for 30 min on days 22–24. Treatment groups were administered tangeretin (25 or 50 mg/kg) orally regularly from days 0 to 14 and on the days of OVA administration, tangeretin was given 2 h prior to each OVA challenge. Tangeretin at the specified doses was dissolved in 1 mL saline and administered orally via gavage. The doses were determined from previous studies in our laboratory. An equal volume of saline was used as control. For a positive control, DEX (2 mg/kg) was injected 1 h prior to OVA administration.

### Collection of bronchoalveolar lavage fluid

Mice (n=6/group) were sacrificed 24 h following the last OVA challenge using an overdose of pentobarbital (50 mg/kg) and a tracheotomy was performed. Bronchoalveolar lavage fluid (BALF) was collected after instillation of ice-cold PBS (0.5 mL) into a lung; with three successive aspirations, a total volume of 1.5 mL was collected via tracheal cannulation. BALF samples were centrifuged (252 *g*, 10 min, 4°C) and the supernatants were stored at –80°C to analyze cytokines. The cell pellet was resuspended in saline (100 µL) and stained with modified Wright-Giemsa stain for 8 min on glass slides. Then the slides were assessed for differential cell counts at ×40 magnification.

### Determination of cytokine levels in BALF

Levels of cytokines (IL-4, IL-5, IL-6, IL-13, IL-17, and IFN-γ) in the BALF were determined by ELISA according to the manufacturers' protocols. For IL-6, IL-17, and IFN-γ, the kits were from R&D Systems (USA).

### Determination of OVA-specific IgE levels in serum and BALF

In serum and BALF, OVA-specific IgE levels were determined as described by Jain et al. (21). Serum was separated from the whole blood that was collected from OVA-induced mice. Briefly, a 96-well microtiter plate coated with OVA (10 mg/mL) was treated with BALF or sera followed by treatment with rat anti-mouse IgE (biotin-conjugated; Pharmingen, USA). The absorbance was read at 405 nm after the addition of avidin-horseradish peroxidase (HRP) solution to each well.

### Isolation of CD4+ T cells

From mice of the different experimental groups, spleens were excised at 24 h after the last OVA challenge. The tissues were placed in Gibco® RPMI 1640 (Thermo Fisher Scientific, USA) and a single-cell suspension of the spleen was prepared. The cells were divided into tubes and washed in phosphate-buffered saline (PBS). CD4+ T cells were isolated by using Mouse CD4 T Lymphocyte Enrichment Set - DM (BD Biosciences, USA) according to the manufacturer's protocol. The two-step negative enrichment method uses the Biotinylated Mouse CD4 T Lymphocyte Enrichment Cocktail that stains erythrocytes and most leukocytes except the CD4 T lymphocytes. The positive and negative fractions were analyzed using flow cytometry (FACSCalibur instrument with CellQuest software; BD Biosciences).

### Flow cytometry analysis of Th17 cells

The spleen cell suspensions were prepared as mentioned above. To analyze the Th17 cell population, cells were incubated with fluorescein isothiocyanate (FITC)-conjugated anti-human CD4 for 30 min at 4°C. After surface staining, the cells were fixed, permeabilized, and stained with PE conjugated-anti-mouse 1L-17A and subjected to flow cytometry (FACSCalibur instrument with CellQuest software; BD Biosciences).

### Measurement of airway hyper-responsiveness

AHR was determined using aerosolized methacholine at different concentrations using Buxco's modular and invasive system (Buxco Electronics Inc., USA). Variation in airway resistance (RI) and lung compliance (Cdyn) after reaction with various concentrations of methacholine were measured as described by Pichavant et al. (22). Briefly, the mice (n=6/group) were anesthetized, tracheotomized, cannulated, and kept within a ventilated body plethysmograph chamber. The animals were monitored closely for a steady baseline airway pressure (<5% variation over 2.5 min) and upon reaching that pressure, they were administered aerosolized PBS or increasing concentrations of methacholine (3.25, 6.25, 12.5, and 25 mg/mL) using a jet nebulizer. Observed RI and Cdyn values are reported as percentages relative to the particular basal values in response to PBS (23).

### Histological examination

Using 10% formalin, lung tissues harvested from mice not used for BALF collection and other analysis (n=6/group) were fixed and then embedded in paraffin wax; 5 μm sections were cut and stained with hematoxylin and eosin (H&E). Peribronchial cell counts were performed according to Duan et al. (24) to assess the degree of leucocyte infiltration. Cell counts were based on a five-point scoring system: 0: no cells, 1: a few cells, 2: a ring of cells 1 cell layer deep, 3: a ring of cells 2–4 cell layers deep, and 4: a ring of cells >4 cell layers deep. To analyze mucus production, periodic acid-fluorescence Schiff stain was used, according to Bao et al. (20). The mucin granules showed red fluorescence at an excitation wavelength of 380-580 nm and were observed at 600-650 nm using a confocal microscope (TCS SP5, Leica Microsystems, USA).

### mRNA levels of cyclinA, cyclinD1, and p27kip1

Cell cycle is tightly regulated by intricate intra-cellular reactions. Cyclin A, cyclin D1 and p27kip1 are some of the chief regulatory molecules of the cell cycle (25). We assessed T-cell proliferation levels. Using RT-PCR cyclinA, cyclinD1, and p27kip1 mRNA levels were determined. From CD4^+^ T lymphocytes, total RNA was isolated using Trizol (Invitrogen, USA). By incubation with reverse transcriptase at 37°C for 1 h, the RNA (1 µg) was reverse transcribed to cDNA. RT-PCR was carried out using specific primers for rat cyclinA, cyclinD1, and p27kip1, as follows: cyclinD1 forward primer 5′-CCTGGATGCTGGAGGTCTG-3′ and reverse 5′-AGGGTGGGTTGGAAATGAAC-3′, cyclinA forward primer 5′-GGGCACCTCGAGGCATTC-3′ and reverse 5′-CCTATTACCCGTCGAGTCTTGAG-3′, p27kip1 forward primer 5′-AGCCTACGCTCCGACTGTTTG-3′ and reverse 5′-CCTCCTGCCACTCGTATCTGC-3′. Relative expression was normalized with GADPH (forward 5′-TCTCCTCTGACTTCAACAGCGAC-3′ and reverse 5′-CCCTGTTGCTGTAGCCAAATTC-3′). PCR was carried as described previously (25).

### Western blot analysis

Isolated CD4^+^ T cells were incubated in lysis buffer with protease inhibitors (0.5 M EDTA, 5 M NaCl, 10% Nonidet P-40, 0.1 M phenylmethylsulfonyl fluoride, 0.1 M EGTA, 1 M sodium fluoride, 1 M HEPES, 2 µg/mL aprotinin, 0.2 M sodium orthovanadate, and 2 µg/mL leupeptin). The concentrations of proteins were determined using a protein assay kit (Bio-Rad Laboratories, USA). The isolated proteins were separated electrophoretically by SDS PAGE (12%). The separated proteins were blotted onto nitrocellulose membranes. Then the membranes were blocked with 5% non-fat dry milk and were incubated with primary antibodies (against cyclinA, cyclinD1, GSK-3β, p-GSK-3β, Akt, p-Akt, mTORc1, PTEN, Notch 1, Jagged 1, Jagged 2, NICD, and p27kip 1) overnight at 4°C and further incubated at room temperature for 1 h with peroxidase-conjugated secondary antibodies. The bands were visualized and analyzed with a chemiluminescence system (Amersham Bioscience, UK). The band intensities were normalized to those of β-actin.

### Statistical analysis

The results are reported as means±SD, from 6 different experiments. Data were evaluated using one-way analysis of variance (ANOVA) followed by Duncan's multiple range test (DMRT) using the SPSS software (ver. 21.0, IBM, USA). P values <0.05 were considered to indicate statistical significance.

## Results

### Tangeretin reduced AHR

**Figure 1.**
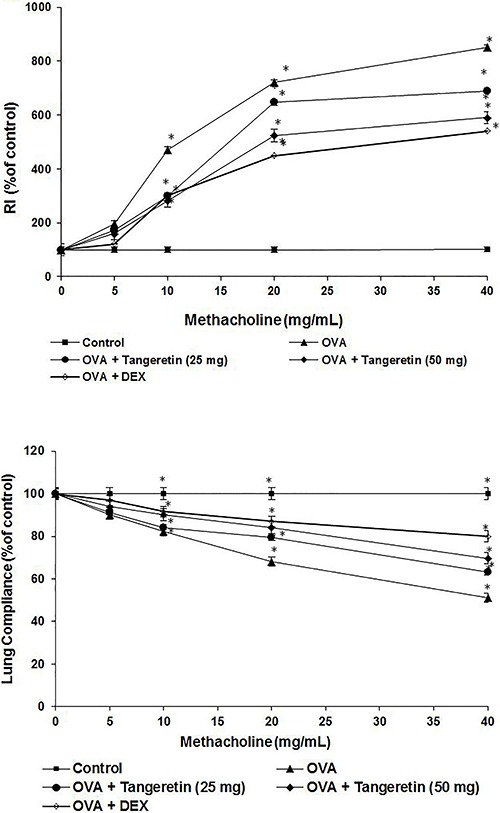
Effects of tangeretin on lung function measured by airway resistance (*top*) and lung compliance (*bottom*). Tangeretin significantly reduced airway resistance and significantly improved lung compliance in ovalbumin (OVA)-induced asthmatic mice. DEX: dexamethasone (positive control). Data are reported as means±SD, n=6. *P<0.05 *vs* control (one way-ANOVA). RI: airway resistance.

The effects of tangeretin (25 and 50 mg/kg) on AHR in reaction to increasing doses of methacholine were determined in terms of RI and Cdyn in mice under mechanical ventilation. AHR in asthma presents with bronchoconstriction in response to various allergic stimuli. RI is defined as the pressure driving respiration divided by the air flow. Cdyn is the distensibility of the lungs and is defined as the change in lung volume caused by a change in lung pressure. We observed that OVA challenge induced AHR, as shown by significantly increased RI values and decreased Cdyn values (Figure 1) versus control mice that were not exposed to OVA. Tangeretin at the doses tested markedly reduced RI and restored Cdyn in response to methacholine (P<0.05). The effects of tangeretin at 50 mg/kg were also positive and were comparable to the positive control, dexamethasone.

### Tangeretin inhibited inflammatory cell infiltration in BALF

**Figure 2.**
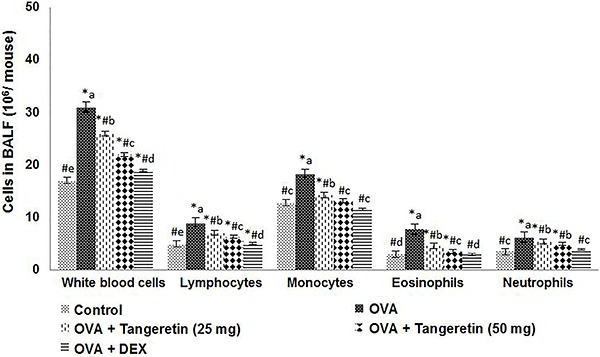
Effects of tangeretin on cell accumulation in bronchoalveolar lavage fluid (BALF). Tangeretin at the doses tested considerably reduced inflammatory cell infiltration into BALF. DEX: dexamethasone. Data are reported as means±SD, n=6. *P<0.05 *vs* control; ^#^P<0.05 *vs* ovalbumin (OVA). P<0.05, different small letters represent significant differences between experimental groups (one-way ANOVA followed by Duncan's multiple range test).

Inflammatory responses are considered a hallmark of allergic asthma. To determine the effects of tangeretin on cell infiltration, changes in total cell levels in BALF were examined. OVA challenge resulted in severe eosinophil and leukocyte infiltration in BALF (Figure 2). Suppression of eosinophil infiltration and a decrease in numbers of monocytes and neutrophils in BALF were observed with tangeretin treatment. The effects of tangeretin were dose-dependent, with the 50 mg/kg dose showing suppressive effects similar to dexamethasone, the positive control.

### Tangeretin reduced OVA-specific IgE levels in serum and BALF

**Figure 3.**
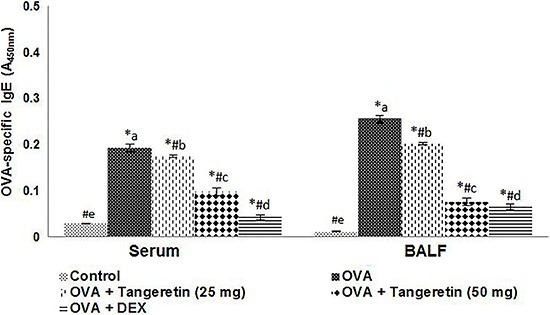
Tangeretin reduced ovalbumin (OVA)-specific IgE levels in serum and bronchoalveolar lavage fluid (BALF). DEX: dexamethasone. Data are reported as means±SD, n=6. *P<0.05 *vs* control; ^#^P<0.05 *vs* OVA. P<0.05, different small letters represent significant differences between experimental groups (one-way ANOVA followed by Duncan's multiple range test).

Expression of allergen-specific IgE in mast cells following allergen challenge is evidence of an early event in asthma (26). OVA-specific IgE levels were determined 24 h after the last OVA challenge in serum and BALF and were higher (P<0.05) than in control mice. A large increase in IgE levels was observed following OVA challenge. Tangeretin treatment in OVA-sensitized mice significantly reduced the IgE levels in serum and BALF (P<0.05; Figure 3). Dexamethasone also caused significant suppression of IgE levels.

### Tangeretin reduced the levels of cytokines in BALF

**Figure 4.**
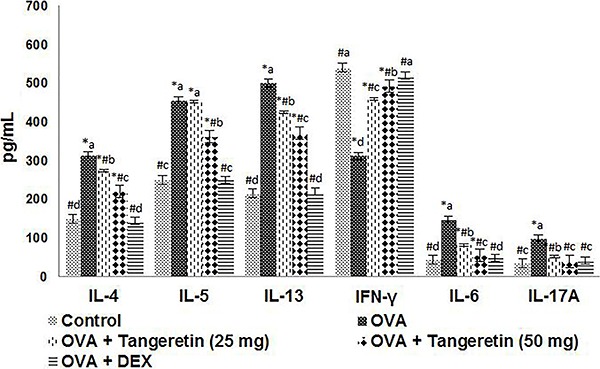
Effects of tangeretin on levels of Th2, Th17, and Th1 cytokines in bronchoalveolar lavage fluid. Tangeretin reduced Th2 and Th17 cytokines whereas it increased the Th1 cytokine IFN-γ. DEX: dexamethasone. Values are reported as mean±SD, n=6. *P<0.05 *vs* control; ^#^P<0.05 *vs* ovalbumin (OVA). P<0.05, different small letters represent significant differences between experimental groups (one-way ANOVA followed by Duncan's multiple range test).

The effects of tangeretin on the secretion of cytokines in BALF were analyzed. The levels of Th2 cytokines (IL-4, IL-5, IL-13) and Th17 cytokines (IL-17A, IL-6) increased following OVA challenge (Figure 4) whereas the levels of the Th1 cytokine IFN-γ decreased. The levels of Th2 and Th17 cytokines decreased, while IFN-γ was increased in mice treated with tangeretin. Furthermore, decreases in Th2 and Th17 cytokines were larger with administration of 50 mg/kg tangeretin than 25 mg/kg. Treatment with dexamethasone showed similar results to that with tangeretin.

### Effects of tangeretin on the Th17 cell population

**Figure 5.**
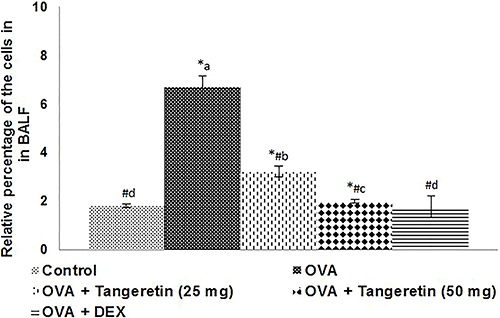
Tangeretin regulates Th17 cell populations in bronchoalveolar lavage fluid (BALF). Data are reported as means±SD, n=6. DEX: dexamethasone. *P<0.05 *vs* control; ^#^P<0.05 *vs* ovalbumin (OVA). P<0.05, different small letters indicate significant differences between experimental groups (one-way ANOVA followed by Duncan's multiple range test).

Th17 cells are involved in allergic responses. The relative proportion of Th17 cells in the spleen was determined by flow cytometry. We saw a markedly increased number of Th17 cells following OVA challenge (P<0.05; Figure 5). The Th17 population was 6.4% after OVA challenge. Administration of tangeretin significantly reduced the population to 3.3 and 1.85%, at the 25 and 50 mg/kg doses, respectively. Thus, effective inhibition of Th17 cells may have contributed to the decreased levels of IL-6 and IL-17A.

### Tangeretin restored lung architecture and reduced mucus hypersecretion following OVA challenge

**Figure 6.**
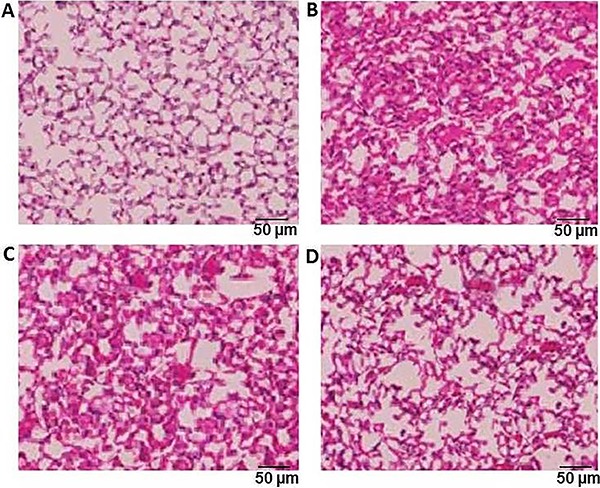
Effects of tangeretin on histopathological changes in lung tissues following ovalbumin (OVA) challenge. Severe cell infiltration was observed after OVA challenge, which was reduced on tangeretin treatment. *A*: Control; *B*: OVA; *C*: OVA + tangeretin (50 mg); *D*: OVA + DEX (dexamethasone). Representative plates were obtained at 400× magnification.

Histological analyses of the lung tissues were conducted to assess changes in lung architecture. Observations from the H&E analysis indicated major infiltration of inflammatory cells into the perivascular and peribronchiolar connective tissues following OVA challenge (Figure 6). Tangeretin treatment at 25 or 50 mg/kg markedly suppressed the infiltration of eosinophils and neutrophils in lung tissues. The increased numbers of eosinophils observed with OVA challenge were decreased. Furthermore, tangeretin restored the lung histology to near normal saline control architecture, with 50 mg/kg tangeretin exhibiting maximum protective effects with negligible cellular infiltration.

**Figure 7.**
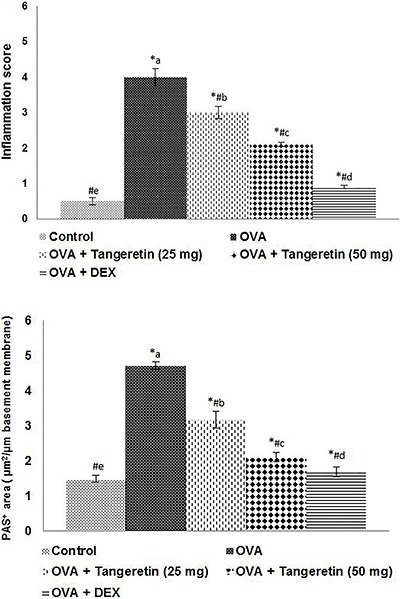
Effects of tangeretin on inflammation score (*top*) and mucus hypersecretion (*bottom*). Data are reported as means±SD, n=6. DEX: dexamethasone; PAS^+^: periodic acid-fluorescence Schiff stain. *P<0.05 *vs* control; ^#^P<0.05 *vs* ovalbumin (OVA). P<0.05, different small letters represent significant differences between experimental groups (one-way ANOVA followed by Duncan's multiple range test).

Allergic asthma is characterized by mucus hypersecretion and AHR. Marked goblet cell hyperplasia along with mucus hypersecretion was observed in OVA-challenged mice that were not treated with tangeretin. A marked reduction in goblet cell hyperplasia was observed with tangeretin treatment (Figure 7). Lung tissue sections treated with the positive control, dexamethasone, did not show mucus hypersecretion and had negligible inflammatory cell infiltration.

### Tangeretin modulated the expression of PI3K signaling and cell cycle proteins

**Figure 8.**
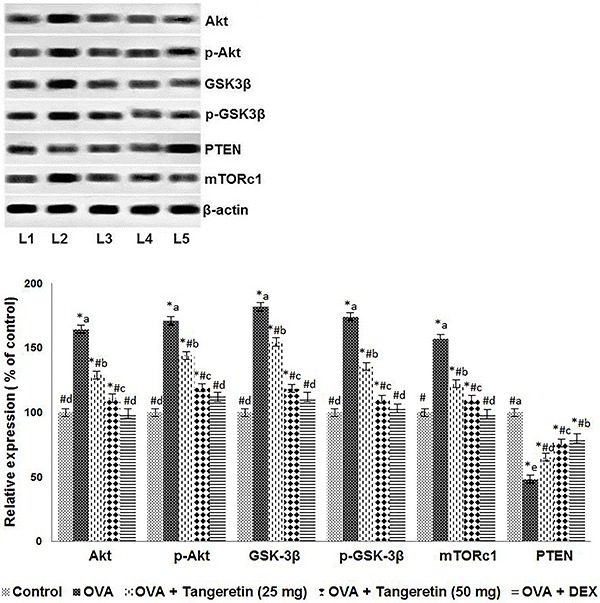
Effect of tangeretin on the expressions of PI3K signaling pathway proteins. L1: Control; L2: OVA; L3: OVA + tangeretin (25 mg); L4: OVA + tangeretin (50 mg); L5: OVA + DEX. Molecular weights: Akt: 55 kDa; p-Akt: 55 kDa; GSK3β: 40 kDa; p-GSK3β: 40 kDa; PTEN: 50 kDa; mTORc1: 250 kDa; β-actin: 40 kDa (*top*). Relative expressions of PI3K/Akt pathway proteins by western blotting (*bottom*). Data are reported as means±SD, n=6. *P<0.05 *vs* control; ^#^P<0.05 *vs* OVA; P<0.05, different small letters represent significant differences between experimental groups (one-way ANOVA followed by Duncan's multiple range test). OVA: ovalbumin; DEX: dexamethasone.

**Figure 9.**
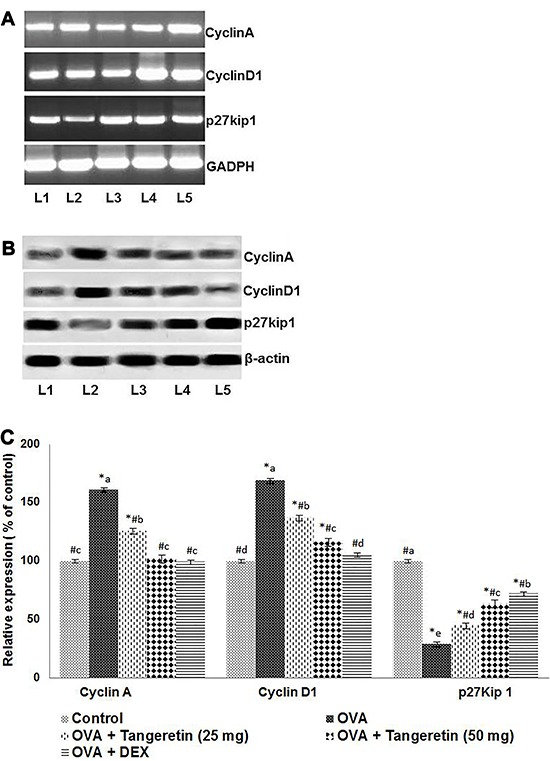
Influence of tangeretin on mRNA expression levels of cell cycle proteins. L1: Control; L2: OVA; L3: OVA + tangeretin (25 mg); L4: OVA + tangeretin (50 mg); L5: OVA + DEX (*A*). Western blot analysis of the expressions of cell cycle proteins. L1: Control; L2: OVA; L3: OVA + tangeretin (25 mg); L4: OVA + tangeretin (50 mg); L5: OVA + DEX (*B*). Molecular weights: CyclinA: 40 kDa; CyclinD1: 30 kDa; p27kip1: 25 kDa; β-actin: 40 kDa. Relative expressions of cell cycle proteins by western blotting (*C*). Data are reported as means±SD, n=6. *P<0.05 *vs* control; ^#^P<0.05 *vs* OVA. P<0.05, different small letters represent significant differences between experimental groups (one-way ANOVA followed by Duncan's multiple range test). OVA: ovalbumin; DEX: dexamethasone.

PI3K plays an important role in T cell signaling. We assessed the influence of tangeretin in PI3K signaling in the isolated CD4 T cells following OVA induction. Increased expression of Akt, p-Akt, GSK-3β, p-GSK-3β, and mTORc1 was observed (Figure 8). The expression of cell cycle proteins, such as cyclinD1 and cyclinA, was upregulated with OVA challenge, whereas the expression of p27kip1, a cyclin-dependent kinase inhibitor, was downregulated (Figure 9). Tangeretin treatment upregulated the levels of PTEN and p27kip1, while downregulating the levels of Akt, GSK-3β, and their phosphorylated forms (Figures 8 and 9). Suppressed expression of cyclinD1 and cyclinA was noted with tangeretin treatment at both the protein and mRNA levels, whereas p27Kip1 mRNA levels increased (Figure 9). Thus, the suppression of cyclins and increased p27kip1 levels suggest suppression of the cell cycle and T cell proliferation. These observations suggest the downregulation of PI3K signaling by tangeretin.

### Tangeretin regulated Notch signaling

**Figure 10.**
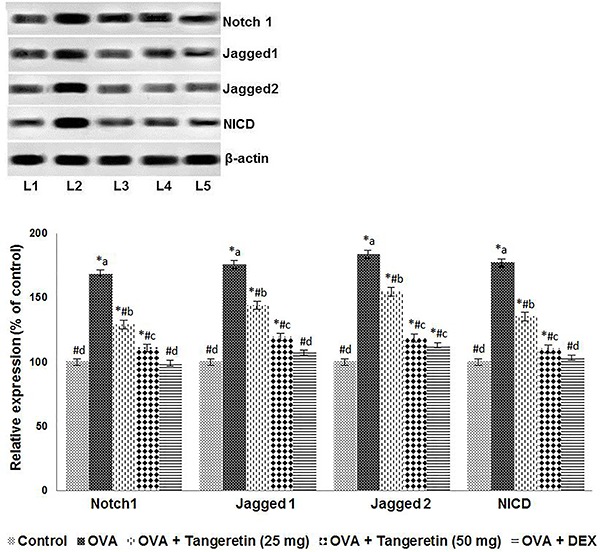
Influence of tangeretin on the proteins of Notch signaling pathways. L1: Control; L2: OVA; L3: OVA + tangeretin (25 mg); L4: OVA + tangeretin (50 mg); L5: OVA + DEX (*top*). Molecular weights: Notch1: 250 kDa; Jagged1: 120 kDa; Jagged2: 125 kDa; NICD: 93 kDa; β-actin: 40 kDa. Relative expressions of Notch pathway proteins by western blotting (*bottom*). Data are reported as means±SD, n=6. *P<0.05 *vs* control; ^#^P<0.05 *vs* OVA; P<0.05, different small letters represent significant differences between experimental groups (one-way ANOVA followed by Duncan's multiple range test). NICD: Notch receptor intracellular domain; OVA: ovalbumin; DEX: dexamethasone.

The Notch signaling pathway is involved in T cell differentiation and activation. OVA challenge resulted in upregulated expression of the Notch 1 receptor, along with its ligands, Jagged 1 and Jagged 2, and the NICD in the isolated CD4+ T cells (Figure 10). These enhanced levels of expression indicate the activation of T cells and their functions in asthma. However, we noted downregulation of Notch signaling in mice that were administered tangeretin at both doses (the 50 mg/kg dose caused more significant suppression of protein levels than 25 mg/kg). This suppression suggests decreased activation of signaling in T cells, resulting in reduced inflammatory responses. These observations indicate the effective downregulation of Notch and PI3K signaling, which could have resulted in the lower levels of cytokines and thus the suppression of immune responses.

## Discussion

Bronchial asthma, a chronic airway disease, is characterized by bronchial hyper-responsiveness, airway inflammation, and remodeling of the airways (27). The important role of T lymphocytes in the pathogenesis of asthma has been documented, and it involves the release of cytokines (28). The CD4^+^ T cell cytokines are associated with cellular infiltration into the airways, mucus hypersecretion, eosinophil accumulation, AHR, and remodeling of the airways and lungs (28,29). AHR accompanied by airway obstruction is characteristic of asthma. Bronchoconstriction in asthma is attributable to hypertrophy of airway smooth muscles and/or contraction and inflammation inside the airways, leading to impaired or reduced lung function (30). AHR reflects the degree of bronchoconstriction. We measured AHR in terms of lung compliance and airway resistance. We found that tangeretin suppressed AHR, improving lung compliance and reducing RI. Tangeretin also mediated reduced inflammation, which may have helped improve lung function.

Infiltration of inflammatory cells, particularly lymphocytes and eosinophils, into the lung and BALF is an important contributor in allergic airway inflammation and considered a hallmark that leads to the development of asthma (31). In line with the previous reports, the lungs of OVA-exposed mice presented with severe inflammatory cell infiltration observed by H&E staining. Mucus hypersecretion with goblet hyperplasia in the airways were also observed with high levels of OVA-specific IgE in BALF and serum. In contrast, tangeretin attenuated inflammatory scores, cellular infiltration, mucus secretion, and restored lung histology to near normal control.

Th2 cytokines (IL-4, IL-5, and IL-13) are known to be critically involved in the pathogenesis of asthma (32). Levels of Th2 cytokines (IL-4, IL-5, IL-13) increased in OVA-exposed asthmatic mice. The cytokine IL-4 is involved in allergic inflammation and airway remodeling (33). IL-5 is associated with eosinophil proliferation, differentiation, survival, recruitment, and activation (34) and IL-13 is involved in the secretion of various proinflammatory factors. Moreover, IL-4, IL-5, and IL-13 are involved in the production of allergen-specific IgE and aid in the release of inflammatory mediators from mast cells (2). Furthermore, the Th17 cytokine IL-17 is involved in eosinophil and neutrophil accumulation and neutrophil activation (4). Thus, the increased levels of these cytokines indicate increased cellular infiltration and inflammation.

We noted suppressed levels of Th2 and Th17 cytokines in the BALF of OVA-induced mice that were administered tangeretin. Furthermore, the reduced levels of IL-17 were consistent with the reduced Th17 cell population. Nevertheless, tangeretin increased IFN-γ levels. These observations reveal decreased inflammatory scores and imply that the infiltration of cells into BALF could be due to the reduced levels of cytokines. The increased levels of the Th1 cytokine IFN-γ observed with tangeretin administration could also have contributed to decreased inflammation.

Ma et al. (35) reported an increase in the effector T cell population in asthma. We also observed increased Th17 cell counts and Th2 and Th17 cytokine levels. The increased cytokine levels could be due to increased expression of cytokines and/or the increased cell population. To assess cell proliferation and the effects of tangeretin, levels of important cell cycle proteins, such as cyclinD1, cyclinA, and the cell cycle inhibitor protein p27kip1, were determined at both the gene and protein levels. CyclinD1 is a key regulator of cell cycle progression, from the G1 to the S phase, whereas cyclinA regulates entry into the M phase from G2 (36). Thus, p27kip1 controls the proliferation of CD4^+^ T cells and their effector function (37). In the present study, increased levels of protein and mRNA for cyclinA and cyclinD1 in the CD4^+^ T lymphocytes of mice induced with OVA indicate increased proliferation of the cells versus the control group. A substantial reduction in p27kip1, at the protein and mRNA levels, was observed, and tangeretin administration led to the downregulation of cyclinD1 and cyclinA and enhanced p27kip1 levels. This suggests the possible inhibitory effects of tangeretin on the irregular proliferation of CD4^+^ T lymphocytes.

The PI3K and Notch signaling pathways have been reported to contribute to the initiation and proliferation of CD4^+^ T cells (14). Zhang et al. (25) reported that CD4^+^ T cell proliferation was regulated by the PI3K and Notch signaling pathways. Consistent with previous reports, we observed increased expression of the Notch1 receptor and its ligands, Jagged 1 and Jagged 2 (25,38). NICD expression also increased. Notch signaling is initiated when Notch receptors bind their ligands, leading to cleavage of NICD, which is then translocated to the nucleus and initiates the transcription of downstream target genes (38). Upregulation of PI3K pathway proteins was also observed in T cells isolated from OVA-alone-induced mice. Nevertheless, significant down-regulation of Notch signaling and PI3K/Akt signaling by tangeretin suggests reduced cell proliferation and survival.

Previous studies have reported that tangeretin exhibits anti-inflammatory effects (14,15,39). Tangeretin was found to inhibit lipopolysaccharide (LPS)-induced nitric oxide, tumor necrosis factor alpha, IL-6, and IL-1β in LPS-stimulated microglia (39). Jang et al. (40) reported that flavonoids tangeretin and nobiletin effectively inhibited NF-κB activation and histamine action following histamine-stimulation in mice. The present study illustrates the potent anti-asthmatic and anti-inflammatory efficiency of tangeretin via reducing inflammatory cytokines and modulating major pathways of inflammatory responses. However, how tangeretin causes these effects and the underlying molecular events need to be explored further.

Collectively, our results indicated the effectiveness of tangeretin in reducing AHR, likely by regulating Th1, Th2, and Th17 cytokine levels and Notch and PI3K signaling. Further experiments with tangeretin could provide a better understanding of the molecular events involved. Thus, tangeretin is a possible candidate for treatment of asthma.
